# Global Estimates of Syphilis in Pregnancy and Associated Adverse Outcomes: Analysis of Multinational Antenatal Surveillance Data

**DOI:** 10.1371/journal.pmed.1001396

**Published:** 2013-02-26

**Authors:** Lori Newman, Mary Kamb, Sarah Hawkes, Gabriela Gomez, Lale Say, Armando Seuc, Nathalie Broutet

**Affiliations:** 1 Department of Reproductive Health and Research, World Health Organization, Geneva, Switzerland; 2 Division of Sexually Transmitted Disease Prevention, Centers for Disease Control and Prevention, Atlanta, Georgia, United States of America; 3 University College London, London, United Kingdom; 4 Amsterdam Institute for Global Health and Development, Amsterdam, The Netherlands; 5 School of Public Health, Imperial College London, London, United Kingdom; Hospital Clinic, Barcelona, Spain

## Abstract

**Background:**

The World Health Organization initiative to eliminate mother-to-child transmission of syphilis aims for ≥90% of pregnant women to be tested for syphilis and ≥90% to receive treatment by 2015. We calculated global and regional estimates of syphilis in pregnancy and associated adverse outcomes for 2008, as well as antenatal care (ANC) coverage for women with syphilis.

**Methods and Findings:**

Estimates were based upon a health service delivery model. National syphilis seropositivity data from 97 of 193 countries and ANC coverage from 147 countries were obtained from World Health Organization databases. Proportions of adverse outcomes and effectiveness of screening and treatment were from published literature. Regional estimates of ANC syphilis testing and treatment were examined through sensitivity analysis. In 2008, approximately 1.36 million (range: 1.16 to 1.56 million) pregnant women globally were estimated to have probable active syphilis; of these, 80% had attended ANC. Globally, 520,905 (best case: 425,847; worst case: 615,963) adverse outcomes were estimated to be caused by maternal syphilis, including approximately 212,327 (174,938; 249,716) stillbirths (>28 wk) or early fetal deaths (22 to 28 wk), 91,764 (76,141; 107,397) neonatal deaths, 65,267 (56,929; 73,605) preterm or low birth weight infants, and 151,547 (117,848; 185,245) infected newborns. Approximately 66% of adverse outcomes occurred in ANC attendees who were not tested or were not treated for syphilis. In 2008, based on the middle case scenario, clinical services likely averted 26% of all adverse outcomes. Limitations include missing syphilis seropositivity data for many countries in Europe, the Mediterranean, and North America, and use of estimates for the proportion of syphilis that was “probable active,” and for testing and treatment coverage.

**Conclusions:**

Syphilis continues to affect large numbers of pregnant women, causing substantial perinatal morbidity and mortality that could be prevented by early testing and treatment. In this analysis, most adverse outcomes occurred among women who attended ANC but were not tested or treated for syphilis, highlighting the need to improve the quality of ANC as well as ANC coverage. In addition, improved ANC data on syphilis testing coverage, positivity, and treatment are needed.

*Please see later in the article for the Editors' Summary*

## Abstract 2

Using multinational surveillance data, Lori Newman and colleagues estimate global rates of active syphilis in pregnant women, adverse effects, and antenatal coverage and treatment needed to meet WHO goals.

## Editors' Summary

**Background:**

Syphilis—a sexually transmitted bacterial infection caused by *Treponema pallidum*—can pass from a mother who is infected to her unborn child. Screening pregnant women for syphilis during routine antenatal care by looking for a reaction to *T. pallidum* in the blood (seropositivity) and then treating any detected infections with penicillin injections has been feasible for many years, even in low-resource settings. However, because coverage of testing and treatment of syphilis remains low in many countries, mother-to-child transmission of syphilis—“congenital syphilis”—is still a global public health problem. In 2007, the World Health Organization (WHO) estimated that there were 2 million syphilis infections among pregnant women annually, 65% of which resulted in adverse pregnancy outcomes: the baby's death during early or late pregnancy (fetal death and stillbirth, respectively) or soon after birth (neonatal death), or the birth of an infected baby. Babies born with syphilis often have a low birth weight and develop problems such as blindness, deafness, and seizures if not treated.

**Why Was This Study Done?:**

In 2007, WHO launched an initiative to eliminate congenital syphilis that set targets of at least 90% of pregnant women being tested for syphilis and at least 90% of seropositive pregnant women receiving adequate treatment by 2015. To assess the initiative's progress and to guide policy and advocacy efforts, accurate global data on the burden of syphilis in pregnancy and on associated adverse outcomes are needed. Unfortunately, even in developed countries with good laboratory facilities, definitive diagnosis of congenital syphilis is difficult. Estimates of the global burden can be obtained, however, using mathematical models. In this study, the researchers generate global and regional estimates of the burden of syphilis in pregnancy and associated adverse outcomes for 2008 using a health services delivery model.

**What Did the Researchers Do and Find?:**

The researchers developed a mathematical model to estimate the number of syphilis-infected pregnant women in each country and in each region, and to estimate the regional and global numbers of adverse pregnancy outcomes associated with syphilis. They used national syphilis seropositivity data and information on antenatal care coverage from WHO and estimates of the effectiveness of screening and treatment from published literature. Using these data and their model, the researchers estimated that, in 2008, 1.4 million pregnant women, 80% of whom had attended antenatal care services, had an active syphilis infection. Assuming a scenario in which the percentage of pregnant women tested for syphilis and adequately treated ranged from 30% for Africa and the Mediterranean region to 70% for Europe (a scenario defined in consultation with WHO advisors), the researchers estimated that maternal syphilis caused 520,000 adverse outcomes in 2008, including 215,000 stillbirths or fetal deaths, 90,000 neonatal deaths, 65,000 preterm or low birth weight infants, and 150,000 infants with congenital disease. About 66% of these adverse effects occurred in women who had attended antenatal care but were either not tested or not treated for syphilis. Finally, the researchers estimated that in 2008, clinical services averted 26% of all adverse outcomes.

**What Do These Findings Mean?:**

These findings, which update and extend previous estimates of the global burden of congenital syphilis, indicate that syphilis continues to affect a large number of pregnant women and their offspring. The current findings, which cannot be directly compared to previous estimates because of the different methodologies used, are likely to be affected by the accuracy of the data fed into the researchers' model. In particular, the data on the percentage of the population infected with syphilis in individual countries used in this study came from the HIV Universal Access reporting system and may not be nationally representative. Nevertheless, these findings suggest that syphilis continues to be an important cause of adverse outcomes of pregnancy, partly because pregnant women often do not receive syphilis screening and prompt treatment during routine antenatal care. The researchers recommend, therefore, that all countries should ensure that all pregnant women receive an essential package of high-quality antenatal care services that includes routine and easy access to syphilis testing and treatment. Congenital syphilis, they conclude, can only be eliminated if decision-makers at all levels prioritize the provision, quality, and monitoring of this basic antenatal care service, which has the potential to reduce infant mortality and improve maternal health.

**Additional Information:**

Please access these websites via the online version of this summary at http://dx.doi.org/10.1371/journal.pmed.1001396.

- The World Health Organization provides information on sexually transmitted diseases, including details of its strategy for the global elimination of congenital syphilis, the investment case for the elimination of mother-to-child transmission of syphilis, and regional updates on progress towards elimination (some information is available in several languages)
- The Pan American Health Organization provides information on efforts to eliminate congenital syphilis in Latin America (in English and Spanish), and the Asia-Pacific Prevention of Parent-to-Child Transmission Task Force provides information on efforts to eliminate congenital syphilis in Asia Pacific
- The US Centers for Disease Control and Prevention has a fact sheet on syphilis (in English and Spanish)
- The UK National Health Service Choices website also has a page on syphilis
- MedlinePlus provides information on congenital syphilis and links to additional syphilis resources (in English and Spanish)
- The London School of Hygiene and Tropical Medicine provides a toolkit for the introduction of rapid syphilis tests
- Haiti: Congenital Syphilis on the Way Out is a YouTube video describing the introduction of rapid diagnostic tests for syphilis in remote parts of Haiti

## Introduction

Syphilis is a severe bacterial disease that in pregnancy may manifest as stillbirth, early fetal death, low birth weight, preterm delivery, neonatal death, or infection or disease in the newborn. Both archaeological and literary evidence suggest that mother-to-child transmission (MTCT) of syphilis, commonly referred to as “congenital syphilis,” is an ancient scourge [1],[2]. In modern times the effectiveness of syphilis testing and treatment in preventing MTCT of syphilis is well-recognized [3]. Diagnosis and prevention of MTCT of syphilis is feasible, inexpensive, and cost-effective in nearly every situation evaluated [4]. Yet, despite the tools being available for over 60 y, MTCT of syphilis persists as a public health problem.

It is unknown exactly what proportion of pregnant women globally receives adequate testing and treatment for syphilis. The World Health Organization (WHO) has begun to monitor syphilis testing and treatment coverage through the HIV Universal Access reporting system, but quality data are not yet available from all countries. In 2011, 63 of 149 low- and middle-income countries reported on coverage of syphilis testing within antenatal care (ANC), with a median of 68% of women in the reporting countries being tested for syphilis at first ANC visit [5]. However, this coverage estimate may be an overestimate of syphilis testing coverage in low- and middle-income countries, as those countries without a functional syphilis screening program are unlikely to have a functional reporting system. A recent multi-country study to assess introduction of rapid point-of-care tests found that ANC syphilis testing at baseline was nonexistent in the Amazonas region of Brazil, 1.7% in Kampala Hospital and rural ANC centers in Uganda, 17.8% in the district hospital and 51 health facilities in Geita District, Tanzania, 51% in a maternity hospital in Lima and ANC centers in Callao, Peru, and 79.9% in Lusaka Hospital and rural ANC centers in Mongu District, Zambia [6]. Other individual country reports have also documented a low coverage of testing and treatment of syphilis in pregnant women. For example, a study in southern China found that 57% of pregnant women were tested for syphilis from 2004 to 2008 [7]. Another study in two provinces in South Africa found that although 71% of pregnant women were tested for syphilis at first ANC visit, only 74% of women who tested positive for syphilis had treatment documented, and only 36% of seropositive women with documented treatment received the recommended three doses of intramuscular penicillin [8].

In 2007, WHO launched its Initiative for the Global Elimination of Congenital Syphilis, with the goal that by 2015 at least 90% of pregnant women are tested for syphilis and at least 90% of seropositive pregnant women receive adequate treatment [4]. The elimination initiative was designed around improving four public health “pillars”: political advocacy and community engagement; adequate coverage and quality of ANC; access to and quality of syphilis testing in ANC; and routine surveillance, monitoring, and evaluation. The regions of the Americas and the Asia Pacific have also launched elimination initiatives, in both cases initiatives integrated with elimination of MTCT of HIV [9],[10].

In order to assess progress in elimination of MTCT of syphilis and to guide policy and advocacy efforts, global data on the burden of syphilis in pregnancy and associated adverse outcomes are needed. Unfortunately, MTCT of syphilis cannot be easily measured globally because definitive diagnosis is difficult, even in developed countries with robust laboratory infrastructure. Thus, estimating the burden of disease must rely on modeled data. In 2007 WHO reported global estimates for congenital syphilis burden based on a review of published data from 1997 through 2003. This work estimated that annually there were 2,036,753 syphilis infections among pregnant women, of which 65% (1,323,889; range: 728,547 to 1,527,565) resulted in adverse pregnancy outcomes [11]. The 1997–2003 estimates were based upon 45 published studies representing 31 countries. All included studies had a sample size of at least 100 women and determined syphilis seropositivity using both treponemal and non-treponemal tests. The 1997–2003 estimates excluded studies from the United States, Canada, and most of Europe because syphilis is rare in these countries and they would not contribute meaningful numbers of congenital syphilis. The 1997–2003 estimates also did not take existing treatment services into account and did not remove background mortality and morbidity (i.e., expected adverse outcomes that would occur in uninfected women).

In order to support the global initiative for elimination of congenital syphilis, WHO has developed 2008 global estimates of maternal syphilis and associated adverse pregnancy outcomes. The 2008 numbers are intended not only to update the earlier 1997–2003 estimates, but also to use a stronger methodology to take into account existing treatment services, remove background mortality, and incorporate data from a greater number of countries. These updated estimates can be used to guide global and regional policy, program, and advocacy efforts to strengthen maternal and child health services and to monitor progress to eliminate MTCT of syphilis.

## Methods

**Figure 1:**
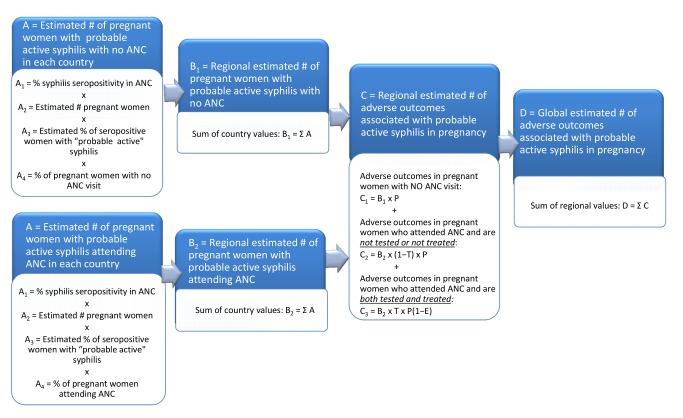
Flowchart of model to estimate number of adverse outcomes associated with syphilis in pregnancy. #, number.

The objective of this analysis was to estimate for 2008 the global and regional number of pregnant women infected with probable active syphilis, as well as the number of associated adverse outcomes of syphilis in pregnancy. This analysis is based upon a health service delivery model that involved four estimation steps (Figure 1): calculation of the number of pregnant women with probable active syphilis in each country, calculation of the number of pregnant women with probable active syphilis in each region, calculation of the number of adverse pregnancy outcomes associated with syphilis in each region, and calculation of the global number of adverse pregnancy outcomes related to syphilis.

Wherever possible, this analysis was done using data provided routinely by countries to United Nations organizations (country-specific data points are available in Dataset S1). Countries were allocated to one of six regions based on WHO regional classifications [12]. For this analysis we defined “probable active syphilis infection” as seropositivity on both treponemal and non-treponemal tests, because women without confirmed test results may have had false positive tests or old or untreated disease that would be unlikely to pose a risk of syphilis transmission to the fetus. We defined “stillbirth” as death of a fetus of at least 28 wk gestation or at least 1,000 g weight, and “early fetal death” as fetal death occurring from 22 to 28 wk gestation (i.e., second and early third trimester); we did not include first trimester losses (miscarriages) [13].

### Step A: Estimate the Number of Pregnant Women with Probable Active Syphilis in Each Country

**Figure 2:**
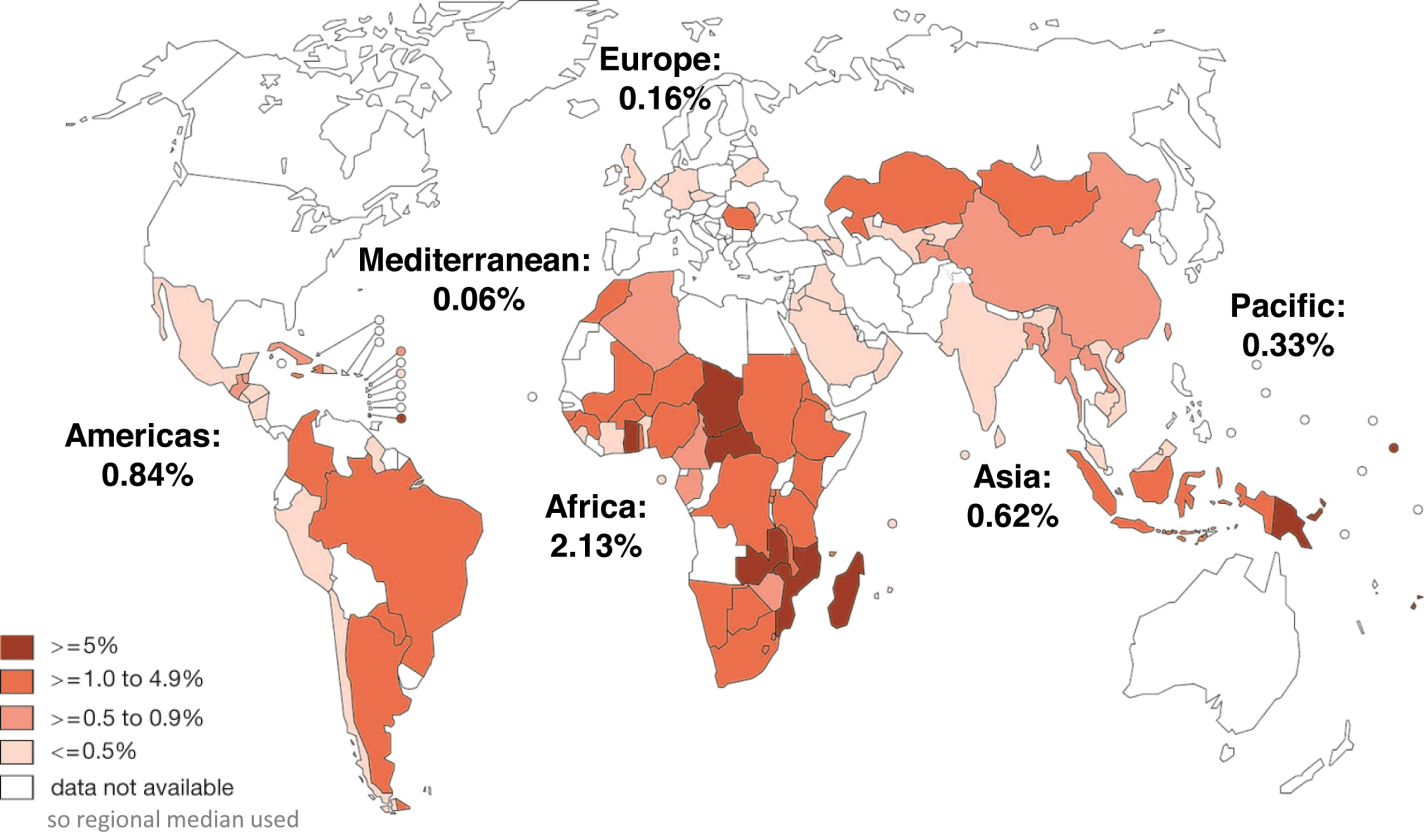
Syphilis seropositivity among antenatal care attendees reported by countries through the WHO HIV Universal Access reporting system in 2008 or 2009, and regional median for non-reporting countries.

For Step A_1_, data on syphilis seropositivity among ANC attendees for each country were those data reported through the WHO HIV Universal Access reporting system for 2008 (data available at the WHO Global Health Observatory Data Repository: http://apps.who.int/gho/data/?theme=main). If 2008 data were not available, we used data reported for 2009 (Figure 2). In 2008 and 2009, data were reported for 97 of 193 countries (50.3%). A regional median was used for countries that did not report data in 2008 or 2009. Countries could report either ANC sentinel survey results or routinely conducted antenatal program data to the HIV Universal Access reporting system. In this reporting system, type of syphilis test used is not reported; thus, it was not possible to distinguish whether reported seropositivity was based upon treponemal, non-treponemal, or combined test results.

The estimated number of pregnant women in Step A_2_ was calculated for each country as the sum of the number of live births per United Nations Population Division estimates for 2008, stillbirths (after 28 wk gestation) per 2008 published estimates, and early fetal deaths (22–28 wk gestation) [14]. Estimates of early fetal deaths are not available for many countries; thus, we estimated these to be approximately 20% of stillbirths based on the early to late fetal death relationship in a limited number of studies mainly in high-income countries [15],[16]. Estimations of fetal deaths did not include first trimester losses (miscarriages).

Step A_3_ included an estimation of the proportion of seropositive pregnant women with probable active syphilis infection. A value of 65% was used as a correction factor to estimate the proportion of seropositive women with both treponemal and non-treponemal syphilis test positivity for the data reported through the HIV Universal Access reporting system. The correction factor was based on data from a literature review of 45 studies on syphilis in pregnancy from 2004 to 2008 [17]. Of these, three studies reported results of treponemal and non-treponemal tests for all pregnant women [18]–[20]. Among all women with either syphilis test positive, the proportion of women with both the treponemal test and the non-treponemal test positive was found to be 63.9% in Mozambique, 61.7% in Bolivia, and 68.0% in Tanzania.

Step A_4_ involved estimating for each country the proportion of pregnant women with and without at least one ANC visit. We used the most recent data for a country available in the WHO Global Health Observatory from 2000 to 2010; data were available for 147 countries [21]. A regional median was used for countries for which data were not available. The four values in Step A were multiplied to obtain the estimated number of pregnant women infected with probable active syphilis attending and not attending ANC in each country.

An approximate uncertainty range for the global number of pregnant women with probable active syphilis was calculated using the delta method and a 10% relative error at the national level for three input variables: syphilis seropositivity, number of pregnancies, and the correction factor for the estimated proportion of women with syphilis with probable active disease.

### Step B: Estimate the Number of Pregnant Women Infected with Probable Active Syphilis in Each Region

Step B involved summing the country values to provide regional estimates of the number of pregnant women with probable active syphilis not attending ANC (B_1_), as well as the number attending ANC (B_2_).

### Step C: Estimate the Number of Adverse Pregnancy Outcomes Associated with Syphilis in Each Region

The objective of Step C was to estimate for each region the total number of adverse pregnancy outcomes related to maternal syphilis. We calculated the number of specific adverse pregnancy outcomes in five categories: any adverse outcome, stillbirth or early fetal death, neonatal death, prematurity or low birth weight, and an infected newborn. Because of the limited availability of data on the proportion of pregnant women tested in ANC and, of those found to be positive, the proportion treated adequately, we devised a simple sensitivity analysis that considered three testing and treatment coverage scenarios defined in consultation with WHO regional advisors: worst, middle, and best case (Table 1). In addition, we considered the scenario proposed as a target for 2015 in the WHO Initiative for the Global Elimination of Congenital Syphilis, in which at least 90% of pregnant women are tested for syphilis at first antenatal visit, and at least 90% of seropositive pregnant women are adequately treated (i.e., testing and treatment coverage *T* = 81% for all regions, except Europe, which was assumed to maintain *T* = 90%).

**Table 1:** Estimated proportion of antenatal care attendees tested and treated in different scenarios for 2008, and proposed Initiative for the Global Elimination of Congenital Syphilis target for 2015.

Region	Worst Case 2008	Middle Case 2008	Best Case 2008	Proposed Syphilis Elimination Target 2015
Africa	10%	30%	50%	81%
Americas	40%	60%	80%	81%
Asia	20%	40%	60%	81%
Europe	50%	70%	90%	90%
Mediterranean	10%	30%	50%	81%
Pacific	40%	60%	80%	81%

If a country tested 80% of all pregnant women, and 80% of those found to be seropositive received appropriate treatment, then *T* = 0.8×0.8 = 64%. The proposed 2015 target for the Initiative for the Global Elimination of Congenital Syphilis is that at least 90% of pregnant women are tested for syphilis and at least 90% of seropositive pregnant women receive treatment (*T* = 0.9×0.9 = 81%). This would apply for all regions except Europe, on the assumption that Europe would be unlikely to provide a lower standard of care than the 2008 best case scenario of *T* = 90%.

In Step C_1_, the total number of adverse outcomes in pregnant women with probable active syphilis who did not attend ANC was calculated as the product of the regional number of infected pregnant women not attending ANC and the proportion *P* of those women expected to have a syphilis-related adverse outcome without treatment. *P* for each adverse outcome category was obtained from a meta-analysis of published clinical literature assessing adverse pregnancy outcomes among women with probable active syphilis without adequate treatment: *P*_any adverse outcome_ = 0.52, *P*_stillbirth/early fetal death_ = 0.21, *P*_neonatal death_ = 0.09, *P*_prematurity/low birth weight_ = 0.06, and *P*_infected newborn_ = 0.16 [22].

We also estimated the number of adverse outcomes in pregnant women with probable active syphilis who attended ANC (Step C_2_ and Step C_3_). Not all women who attend ANC are tested for syphilis and adequately treated if seropositive. Therefore, we estimated adverse outcomes for women who were both tested and treated (*T*) (Table 1), as well as for those who were not (1−*T*). In women who are not both tested and treated, the number of adverse outcomes is the number not tested and treated multiplied by the proportion expected to have an adverse outcome (*P*). Even among women who are both tested and treated, a small number of adverse events can be expected, particularly if syphilis cases are identified late in pregnancy. A systematic review by Blencowe et al. found that the effectiveness *E* of screening and treatment with penicillin in reducing adverse outcomes was as follows: *E*_neonatal death_ = 0.80, *E*_prematurity/low birth weight_ = 0.64, and *E*_infected newborn_ = 0.97 [23]. For this analysis, *E*_stillbirth/early fetal death_ = 0.82 was based on the estimate of effectiveness for stillbirth alone, and *E*_any adverse outcome_ = 0.84 was based on the weighted mean of *E*_neonatal death_, *E*_prematurity/low birth weight_, *E*_infected newborn_, and *E*_stillbirth/early fetal death_, using as weights the proportion of women with the corresponding adverse outcome.

### Step D: Estimate the Global Number of Adverse Pregnancy Outcomes Related to Syphilis

Step D summed the regional estimates for each adverse outcome to produce a global estimate of the number of adverse pregnancy outcomes related to syphilis.

Finally, we calculated the number of adverse pregnancy outcomes averted by current services by subtracting the number of adverse outcomes in the middle case testing-and-treatment-coverage scenario from a baseline number of adverse outcomes estimated in the absence of any services.

## Results

We estimated that in 2008 there were 1,360,485 (range 1,160,195–1,560,776) pregnant women with probable active syphilis infections, and of these women, 1,085,637 (79.8%) attended ANC. The estimated number of infected pregnant women by region was 535,203 in Africa (39.3%), 106,500 in the Americas (7.8%), 603,293 in Asia (44.3%), 21,602 in Europe (1.6%), 40,062 in the Mediterranean (3.0%), and 53,825 (4.0%) in the Pacific. Without any screening or treatment services, these pregnant women with probable active syphilis would have had 707,452 adverse pregnancy outcomes, including 285,702 stillbirths or early fetal deaths, 122,444 neonatal deaths, 81,629 premature or low birth weight infants, and 217,678 infected newborns. Thus, in the absence of any antenatal syphilis screening and treatment, an estimated 408,146 fetal or perinatal deaths and 299,307 infants at risk for early death would have resulted from maternal syphilis infection.

**Figure 3:**
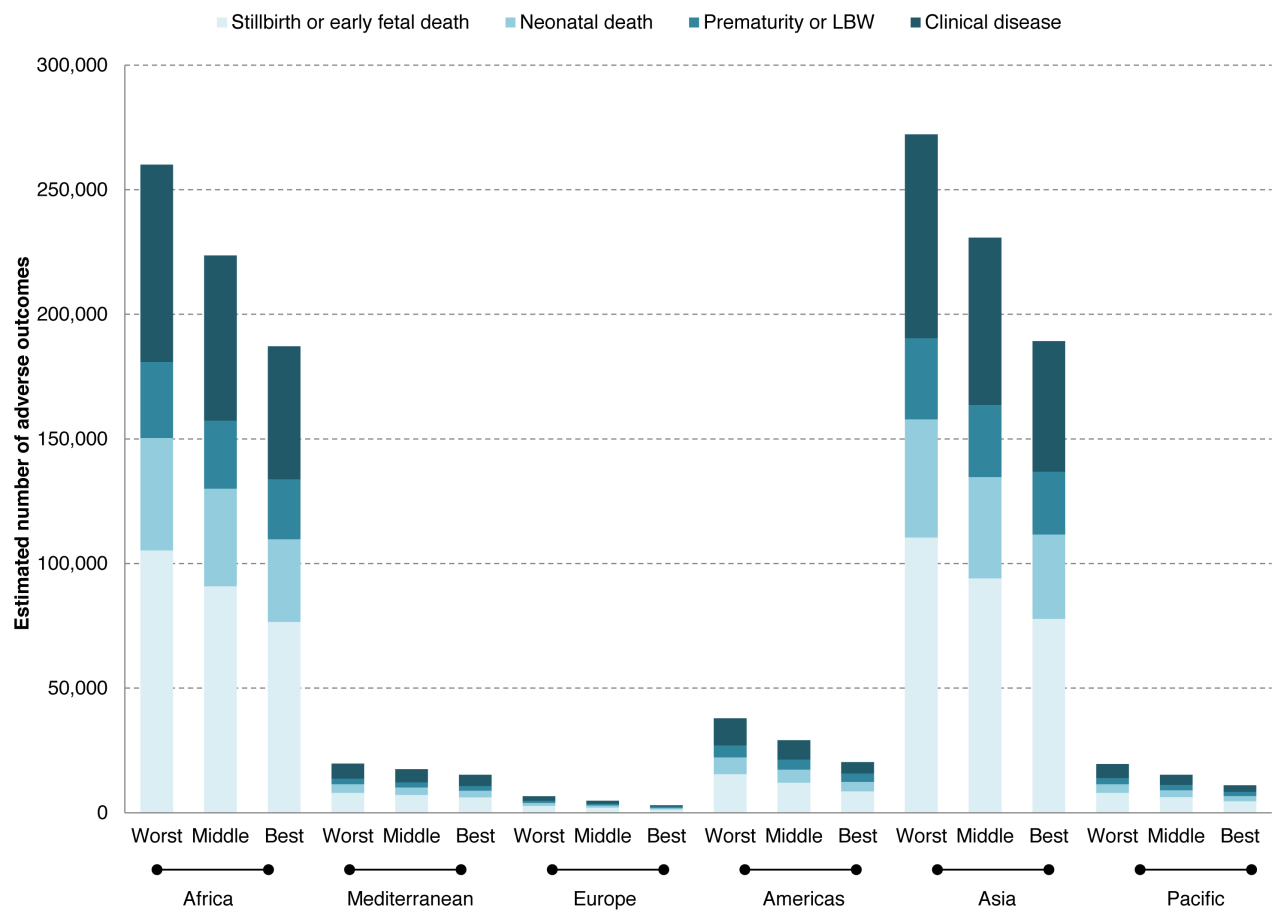
Estimated number of adverse outcomes associated with syphilis in pregnancy in a worst, middle, and best case scenarios of testing and treatment in 2008. LBW, low birth weight.

Figure 3 shows the estimated number of adverse outcomes in 2008 associated with syphilis globally and by region for the worst, middle, and best case scenarios of testing and treatment of women who are seropositive. Considering the middle case scenario, we estimated that globally there were 520,905 (best: 425,847; worst: 615,963) adverse outcomes associated with syphilis in pregnancy, which included 212,327 (best: 174,938; worst: 249,716) stillbirths or early fetal deaths, 91,764 (best: 76,141; worst: 107,397) neonatal deaths, 65,267 (best: 56,929; worst: 73,605) preterm or low birth weight infants, and 151,547 (best: 117,848; worst 185,245) infected newborns. In summary, globally in 2008 in a middle case scenario, untreated maternal syphilis resulted in approximately 304,091 fetal or perinatal deaths and 216,814 syphilis-infected infants at risk for early death. Examination of any adverse outcome by region showed that the majority of adverse outcomes (87%) were in Africa and Asia. Of note, 66% of all adverse outcomes occurred in women who had attended ANC.

In 2008, using the middle case scenario for antenatal screening and treatment, approximately 186,548 (26%) of all adverse outcomes of syphilis in pregnancy were averted. Specifically, the averted outcomes were 73,375 stillbirths or early fetal deaths, 30,679 neonatal deaths, 16,362 preterm or low birth weight infants, and 66,131 infected newborns (Table 2). By region, the number of all adverse outcomes averted in 2008 in the middle case scenario was estimated as 54,676 in Africa, 26,318 in the Americas, 82,949 in Asia, 6,447 in Europe, 3,398 in the Mediterranean, and 12,758 in the Pacific. If the elimination initiative targets for 2015 of at least 90% testing and 90% treatment had been reached in 2008, then 385,815 (55%) of all adverse outcomes could have been averted: 151,753 stillbirths or early fetal deaths, 63,451 neonatal deaths, 33,840 preterm or low birth weight infants, and 136,771 infected newborns.

**Table 2:** Estimated number of adverse outcomes associated with syphilis in pregnancy averted in the middle scenario in 2008, and if Initiative for the Global Elimination of Congenital Syphilis targets for 2015 for testing and treatment had been met in 2008.

Region	Stillbirth or Early Fetal Death		Neonatal Death		Prematurity or Low Birth Weight		Infected Newborn		Any Adverse Outcome	
	Middle Scenario	ECS Target	Middle Scenario	ECS Target	Middle Scenario	ECS Target	Middle Scenario	ECS Target	Middle Scenario	ECS Target
Africa	21,506	58,066	8,992	24,278	4,796	12,948	19,383	52,333	54,676	147,626
Americas	10,352	13,975	4,328	5,843	2,308	3,116	9,330	12,595	26,318	35,530
Asia	32,627	66,069	13,642	27,625	7,276	14,733	29,406	59,546	82,949	167,973
Europe	2,536	3,261	1,060	1,363	566	727	2,286	2,939	6,447	8,290
Mediterranean	1,336	3,608	559	1,509	298	805	1,204	3,252	3,398	9,174
Pacific	5,018	6,775	2,098	2,833	1,119	1,511	4,523	6,106	12,758	17,224
Global total	73,375	151,753	30,679	63,451	16,362	33,840	66,131	136,771	186,548	385,815

ECS, Initiative for the Global Elimination of Congenital Syphilis.

## Discussion

Globally, nearly 1.4 million pregnant women in 2008 had probable active syphilis infection and were at risk of transmitting the disease perinatally to their unborn children. This is lower than previously reported 1997–2003 WHO estimates of approximately 2 million pregnant women annually with untreated syphilis infection, and suggests some progress may have been made over the past decade in syphilis prevention and control. However, the extent of progress is difficult to determine, as the estimates used differing methodological approaches. In comparison to the 1997–2003 estimates, the 2008 estimates were intended to take into account existing treatment services, remove background mortality and morbidity unrelated to syphilis, and better account for the anticipated increasing availability of testing and treatment over time. In addition, because of the paucity of published cross-sectional survey data since 2003 on syphilis in pregnancy, the 2008 estimates were based on data reported to WHO routinely by countries through the HIV Universal Access reporting system rather than on data from a literature review.

Despite differences in methodology, both the 1997–2003 and the 2008 estimates indicate that syphilis in pregnancy continues to be an important cause of mortality and morbidity in pregnancy. This is unsettling given the fact that universal syphilis screening in ANC and prompt treatment of women testing positive are basic interventions that have been proven to be cost-effective even in low prevalence settings. Additionally, rapid point-of-care syphilis tests allowing testing and treatment in almost any clinical setting were not widely available in 2003, but certainly should have been by 2008. These 2008 estimates support that countries in every region of the world should scale up screening and treatment for syphilis in pregnancy, and that doing so could substantially reduce preventable perinatal death and disability.

Our estimates suggest that over 520,000 adverse pregnancy outcomes due to syphilis occurred in 2008, of which approximately 215,000 were stillbirths or early fetal deaths, 90,000 were neonatal deaths, 65,000 were premature or low birth weight infants, and another 150,000 were infected newborns. Our estimates do not include additional deaths that would be expected to occur after the first month of life due to prematurity, low birth weight, or congenital infections, estimated in one study to result in approximately 10% mortality by 1 y (i.e., approximately 21,500 additional infant deaths) [24].

Approximately one-fifth (20%) of all pregnant women with syphilis did not attend ANC. Thus, efforts to ensure universal access to early ANC are fundamental in eliminating congenital syphilis, as well as other causes of preventable infant mortality. But importantly, our data suggest that two-thirds of the adverse outcomes due to syphilis occurred in women who had attended ANC at least once, but either were not screened or, if they were screened and tested positive, did not receive appropriate treatment with intramuscular benzathine penicillin. The vast majority of outcomes that occurred in 2008 could have been prevented had the women received quality early ANC that included syphilis testing and access to effective therapies, as recommended by WHO. Syphilis testing and treatment are relatively inexpensive compared with other interventions, with tests typically costing less than US$1, and treatment slightly less than that. To reduce cases of congenital syphilis, it will be important to incorporate syphilis testing and treatment into routine procurement and distribution systems to ensure that pregnant women receive an essential minimal package of ANC interventions.

Existing health care services were able to avert one out of every four expected adverse outcomes in 2008. However, if the proposed testing and treatment targets for 2015 outlined in the Initiative for the Global Elimination of Congenital Syphilis had been reached in 2008, over half of all expected adverse outcomes could have been averted. Further research is needed to define how many cases and what level of service delivery is needed to attain the ultimate goal of “elimination of congenital syphilis as a public health problem.” However, given that screening and treatment for preventing MTCT of syphilis is not 100% effective, primary prevention of syphilis in pregnant women is also an important strategy that needs to be addressed to truly eliminate congenital syphilis.

While substantial progress has been made in the utilization of ANC (in 2009 WHO estimated that approximately 81% of all pregnant women had attended at least one ANC visit) [25], congenital syphilis still occurred for a variety of reasons: many of these visits were too late to avert an adverse outcome, clinics may not have offered testing, testing may not have been affordable, women may not have followed up or received their test results, treatment may not have been available, or treated women may have been reinfected by untreated sexual partners [26].

Our estimates are subject to some limitations. First, there are no global data on estimated numbers of early fetal deaths; national estimates of early fetal deaths had to be crudely extrapolated from estimated numbers of stillbirths using a correction factor based mainly on data from high-income countries. Improved data on early fetal death, in particular in low- and middle-income countries, is necessary to understand the full extent of the impact of this limitation.

In addition, these estimates rely on data reported through the HIV Universal Access reporting system and do not include separate published studies of syphilis seropositivity, as was previously done for the 1997–2003 estimates. The advantage of HIV Universal Access data is that they are reported by most developing countries on a routine basis, involve a larger sample size than most published studies, and are less subject to a publication bias. However, several countries did not report through the HIV Universal Access system, including some high-income countries in North America, Europe, and the Mediterranean with low syphilis seropositivity, as well as some particularly low resource countries without organized screening programs and with high syphilis seropositivity. It is unclear how this underreporting would affect the overall estimates.

Although countries are asked to report nationally representative data, it is possible that these data are over-representative of urban populations with greater access to syphilis prevention and treatment services than rural populations. If syphilis seropositivity is higher in rural settings (and programs are less effective), our calculations may underestimate the true burden of syphilis-associated adverse outcomes.

In addition, HIV Universal Access data do not include information on testing methodologies, test titer, stage of disease, or treatment history, and therefore it is unclear whether or not a country's data includes women with latent or previously treated disease. This analysis applied a correction factor for “probable active” disease to the entire dataset to account for this; however, adjustment may not have been necessary in all cases and may have led to underestimation of the true burden of active disease.

Because of a lack of representative data on current testing and treatment coverage, these estimates relied on expert opinion of current testing and treatment coverage, and the experts may have miscalculated. In order to explore the validity of these estimates, we present a basic sensitivity analysis of testing and treatment coverage to account for the uncertainty related to this approximation. Although WHO is working to improve collection of data on syphilis testing and treatment through the HIV Universal Access reporting system, these testing and treatment data are not yet felt to be accurate and representative at a global level (the highest performing countries are more likely to report service delivery indicators). Thus, more work with countries to strengthen surveillance and monitoring systems is needed.

Finally, these estimates do not account for the timing of treatment of syphilis in pregnancy. Although syphilis transmission has been documented to occur very early in pregnancy, existing data suggest that catastrophic outcomes due to syphilis require development of the fetal immune system (i.e., after 18–20 wk gestation) [27]. It is also recognized that the effectiveness of screening and treatment is lower in the third trimester than in the first and second trimesters [28].

The limitations of these estimates highlight the urgent need for improved data through stronger national surveillance and monitoring systems. All countries should know at least the three core indicators related to MTCT of syphilis in their population: what proportion of ANC attendees are tested for syphilis, what proportion are seropositive, and what proportion of syphilis seropositive ANC attendees are adequately treated [29]. WHO hopes to work with countries to improve capacity to monitor and report on these three core indicators through the WHO HIV Universal Access reporting system. Better national data are also needed on how early pregnant women seek care and how many women have early fetal deaths, as well as how estimates of adverse outcomes such as congenital syphilis and stillbirth attributable to syphilis compare to actual reported cases of congenital syphilis and stillbirth attributable to syphilis.

In summary, this analysis indicates that syphilis continues to be an important cause of adverse outcomes of pregnancy, including substantial numbers of perinatal deaths and disabilities. Given that an increasing proportion of the infant mortality that Millennium Development Goal 4 aims to address by 2015 occurs during the first month of life, investing in elimination of MTCT of syphilis is a low-hanging fruit for reducing neonatal mortality, as well as stillbirths. Primary prevention of syphilis in people of reproductive age is an important first step towards reducing these deaths. Better data are needed to raise local awareness of the burden of syphilis in pregnancy, an old scourge that is often overlooked in modern day public health programs. Countries also need to ensure that quality-assured syphilis testing is available in all ANC settings, now possible even in remote care settings with the introduction of rapid point-of-care diagnostics. In addition, efforts are needed to ensure universal access to early ANC, as well as improved quality of ANC, so that all pregnant women receive an essential package of services that includes routine and early access to point-of-care testing and adequate treatment for syphilis if seropositive. MTCT of syphilis can only be eliminated if decision-makers at all levels prioritize the provision and quality of this basic ANC service.

Elimination of MTCT of syphilis directly supports attainment of Millennium Development Goals 4, 5, and 6 through reduction in infant mortality, improved maternal health, and primary prevention of HIV [30]. With just a short amount of time left to achieve the Millennium Development Goals, the United Nations Secretary-General launched the Global Strategy for Women's and Children's Health, which calls for improved coordination around maternal and newborn health issues [31]. Bringing ministries of health and partners together to provide universal coverage of antenatal syphilis screening and to ensure treatment of all pregnant women infected with syphilis is a specific example of an activity that would greatly support the Global Strategy for Women's and Children's Health.

## Supporting Information

Dataset S1 **Country-level data and assumptions used for 2008 estimates of syphilis in pregnancy and associated adverse outcomes.**
(XLSX)

## Abbreviations

ANC: antenatal care
MTCT: mother-to-child transmission
WHO: World Health Organization
